# Telomerase prevents accelerated senescence in glucose-6-phosphate dehydrogenase (G6PD)-deficient human fibroblasts

**DOI:** 10.1186/1423-0127-16-18

**Published:** 2009-02-05

**Authors:** Yi-Hsuan Wu, Mei-Ling Cheng, Hung-Yao Ho, Daniel Tsun-Yee Chiu, Tzu-Chien V Wang

**Affiliations:** 1 Graduate Institute of Basic Medical Sciences, Chang Gung University, Kwei-San, Tao-Yuan 333, Taiwan; 2 School of Medical Biotechnology, Chang Gung University, Kwei-San, Tao-Yuan 333, Taiwan; 3 Department of Molecular and Cellular Biology, Chang Gung University, Kwei-San, Tao-Yuan 333, Taiwan

## Abstract

Fibroblasts derived from glucose-6-phosphate dehydrogenase (G6PD)-deficient patients display retarded growth and accelerated cellular senescence that is attributable to increased accumulation of oxidative DNA damage and increased sensitivity to oxidant-induced senescence, but not to accelerated telomere attrition. Here, we show that ectopic expression of hTERT stimulates telomerase activity and prevents accelerated senescence in G6PD-deficient cells. Stable clones derived from hTERT-expressing normal and G6PD-deficient fibroblasts have normal karyotypes, and display no sign of senescence beyond 145 and 105 passages, respectively. Activation of telomerase, however, does not prevent telomere attrition in earlier-passage cells, but does stabilize telomere lengths at later passages. In addition, we provide evidence that ectopic expression of hTERT attenuates the increased sensitivity of G6PD-deficient fibroblasts to oxidant-induced senescence. These results suggest that ectopic expression of hTERT, in addition to acting in telomere length maintenance by activating telomerase, also functions in regulating senescence induction.

## Background

Normal human cells grown *in vitro *replicate for a limited period of time before entering senescence [1], a term that has been used primarily to describe a signal transduction pathway that leads to the irreversible growth arrest of cells in culture. Among the various stimuli that are known to trigger senescence [2,3], telomere attrition and oxidative damage (produced during normal cellular proliferation) are the basis for the "telomere hypothesis" [4,5] and "free radical theory" [6,7], respectively, postulated to account for the aging process.

According to the "telomere hypothesis" of aging, dysfunctional telomeres caused by telomere attrition are postulated to initiate the senescent phenotypes. Telomere attrition occurs because the ends of linear chromosomal DNA cannot be completely replicated by normal DNA polymerases, and, therefore, telomere DNA becomes shortened with each round of DNA replication [8,9]. When telomeres reach a critical length, they become dysfunctional and trigger so-called replicative senescence. Activation of telomere length-maintenance mechanisms, such as expression of telomerase, a specialized reverse transcriptase that synthesizes telomeric DNA repeats at chromosome ends, is thought to counteract replicative senescence [5,10,11]. In support of this postulate, normal human somatic cells express low or undetectable telomerase activity and are mortal. In contrast, a majority of immortal and cancer cells have an indefinite proliferative capacity and maintain their telomere length by upregulating telomerase [12,13]. Ectopic expression of telomerase has been shown to extend the lifespan of many normal human cells cultured *in vitro *[14-16].

The free radical theory of aging postulates that the accumulation of oxidative damage is the central mediator of the aging process [6,7]. Reactive oxygen species produced during normal cellular metabolism or from exogenous sources, such as drugs and radiation, are known to react with biomolecules, including proteins and DNA. It is this accumulation of oxidative damage over time that is postulated to trigger senescence [6]. In support of this hypothesis, exposure to oxidative stress (e.g., *tert*-butyl hydroperoxide, hydrogen peroxide, or a hyperbaric atmosphere with high O_2 _partial pressure) triggers senescence, and oxidative DNA damage is known to accrue during senescence [17-23]. In addition, growth of cells under hypoxic conditions (3% [v/v] O_2 _instead of the normal atmospheric O_2 _level) is known to delay cellular senescence of fibroblasts [24].

Despite supporting evidence for both of these theories, based on multiple experimental approaches, little is known about the relative roles of these two mechanisms in the induction of senescence or the potential interactions of the signaling pathways they trigger. Recently, we have shown that fibroblasts derived from glucose-6-phosphate dehydrogenase (G6PD)-deficient patients displayed retarded growth and accelerated cellular senescence [25]. Evidence indicates that the accelerated cellular senescence observed in G6PD-deficient cells arises because of increased accumulation of oxidative DNA damage and an increased sensitivity to oxidant-induced senescence, but not to accelerated telomere attrition [26]. These data indicate that G6PD status – and thus proper redox balance – is a determinant of cellular senescence. It is not yet known, however, whether increased oxidative DNA damage is the only important determinant of senescence induction in G6PD-deficient cells. To address this point, we asked whether activation of telomerase activity might be capable of overcoming the accelerated senescence observed in such cells. Here, we present evidence that ectopic expression of hTERT, the key regulator of telomerase, activates telomerase activity and prevents premature senescence in G6PD-deficient cells.

## Materials and methods

### Chemicals, enzymes and oligonucleotides

Dulbecco's modified Eagle's medium (DMEM), trypsin, penicillin, streptomycin and amphotericin were purchased from Gibco (Karlsruhe, Germany). The anti-G6PD antibody was from Genesis Biotech (Taiwan). The anti-actin antibody was from Santa Cruz Biotechnologies (Santa Cruz, CA, USA). The antibiotic, G418 sulfate, was from Promega (Madison, WI, USA). The TeloTAGGG assay kit, alkaline phosphatase and protein kinase K were from Roche (Roche, Mannheim, Germany). Taq DNA polymerase was from Qiagen. The sequence and source of TS and CX oligonucleotides have been described [27].

### Cell culture

Normal human fibroblasts (HFF3) and G6PD-deficient fibroblasts (HFF1) were routinely cultured in DMEM supplemented with 10% fetal bovine serum, 100 units/ml penicillin, 100 units/ml streptomycin and 0.25 mg/ml amphotericin at 37°C in a humidified atmosphere containing 5% CO_2_. Human fibrosarcoma cells (HT1080), used as a positive control in soft agar and telomerase assays, were from American Type Culture Collection (ATCC). BOSC23 and PT67 cells were used for preparation of retroviral particles as previously described [26].

### Plasmids, retroviral packaging and infection

The plasmid, pBABE-Puro-hTERT [28], a retroviral construct that expresses hTERT, was kindly provided by Dr. E. Blackburn. Retroviral packaging and infection were accomplished as previously described [26], with the exception that the retrovirus producer cells and hTERT-expressing clones were selected in a medium containing 2 μg/ml puromycin.

### Telomerase activity assay

A PCR-based telomeric amplification protocol (TRAP) was used to assay telomerase activity. The preparation of cell extracts, the PCR amplification conditions, and the analysis of PCR products by electrophoresis on polyacrylamide gel were as described [29].

### Soft agar assay

To assay for contact-independent growth in soft agar, cells were trypsinized and resuspended at 3.3 × 10^3 ^cells/ml in supplemented DMEM containing 0.35% melted agarose. Aliquots (1.5 ml) of cell suspension were poured into 10-cm Petri dishes pre-solidified with 1.5 ml 0.5% agarose in supplemented DMEM. The immobilized cells were grown for 14 days in a humidified chamber at 37°C with 5% CO_2_. Colonies were then photographed and counted.

### Karyotyping of hTERT-expressing cells

Cytogenetic examination was performed with hTERT-expressing cells at passage 11–14. After incubating with 0.06 μg/ml colcemid for 16 h to arrest cells in mitosis, fibroblasts were collected by trypsinization and fixed with Carnoy's fixative (a 3:1 mixture of methanol and glacial acetic acid). Fixed fibroblasts were placed on a glass slide, air-dried and stained with a 6% Giemsa solution for 3.5 min. The number of chromosomes in at least 40 cells was analyzed from each fibroblast preparation.

### Assay for telomere length

Cell pellets were lysed in 1 M Tris-EDTA buffer (pH 7.4) containing 0.5% SDS, and treated with 200 μg/ml proteinase K for 18 h at room temperature. Genomic DNA was then isolated by phenol-chloroform extraction. Genomic DNA was digested with *HinfI *and *RsaI*, and DNA fragments were separated by electrophoresis in 0.8% agarose gels. The DNA fragments containing telomeric repeats were identified by Southern blotting using a *Telo*TAGGG telomere-length assay kit (Roche, Mannheim, Germany). Average telomere fragment length (TFR) was determined from measurements of the intensity of chemiluminescent signals at each molecular mass, obtained by scanning the radiogram using a Molecular Dynamics Personal Densitometer (Sunnyvale, CA, USA). Signal densities were analyzed with ImageQuaNT software (Molecular Dynamics), and mean TRF lengths were calculated according to manufacturer's recommendation.

### Assay for H_2_O_2_-induced premature senescence

Premature senescence was tested in fibroblasts treated with different concentrations of H_2_O_2 _for 1.5 h using senescence-associated (beta)-galactosidase (SA-β-gal) as a biomarker of senescence. After H_2_O_2 _treatment, the medium was replaced with fresh complete medium, and cells were cultured for 72 h before staining for SA-β-Gal activity as previously described [26]. Quantification of premature senescence was determined by calculating the rate of conversion of 4-methylumbellliferyl-β-D-galactopyranoside (MUG) to the fluorescent product, 4-methylumbelliferone (4-MU), at pH 6.0 as previously described [30]. The relative increase in 4-MU fluorescence per mg protein was determined by subtracting the untreated control values from the H_2_O_2_-treated values.

## Results

### Ectopic expression of hTERT immortalizes fibroblasts derived from normal and G6PD-deficient patients

**Figure 1:**
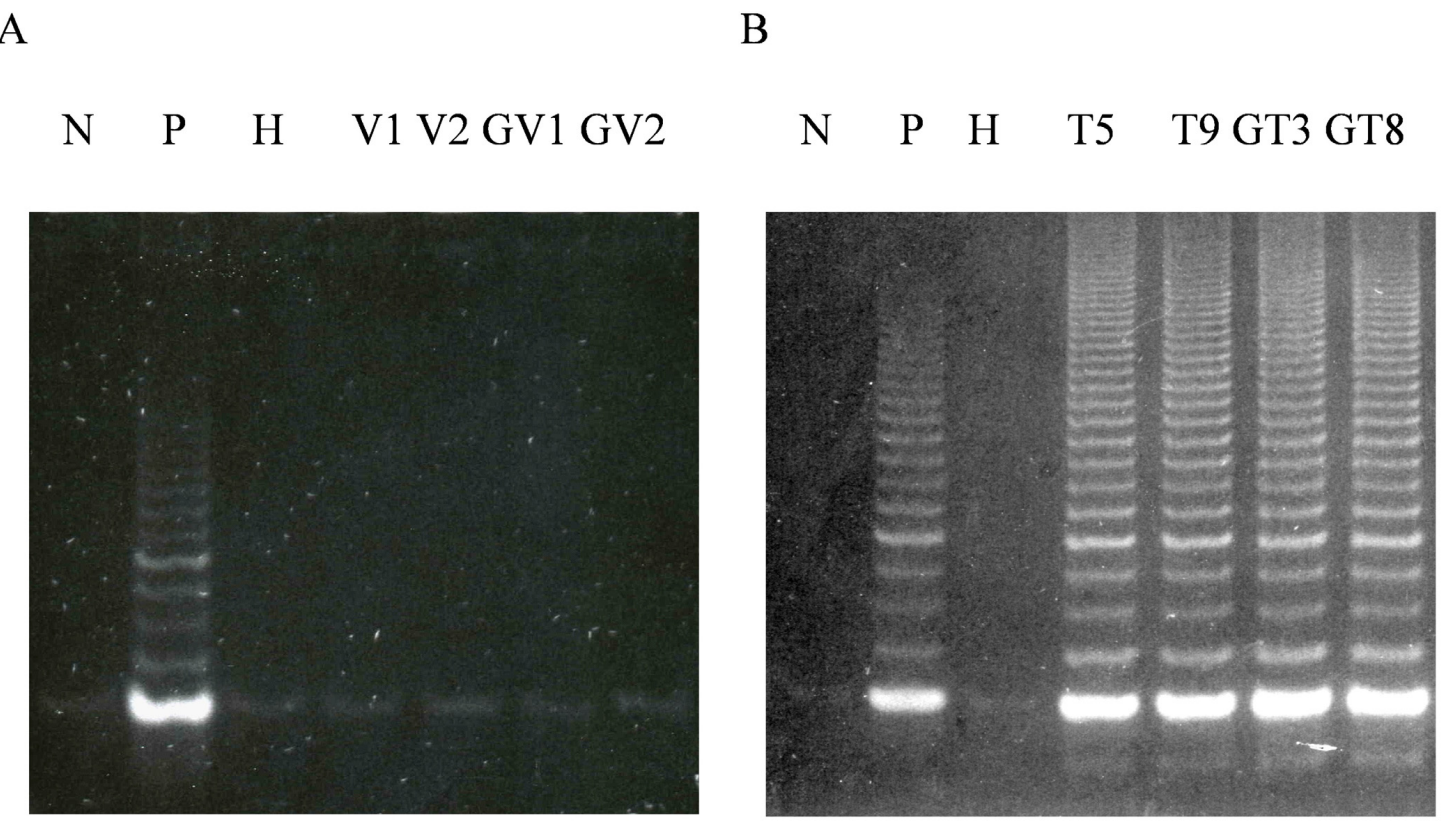
**Telomerase activity of hTERT-transfected normal and G6PD-deficient fibroblasts**. Normal fibroblasts HFF3 and G6PD-deficient fibroblasts HFF1 were infected with retroviral vector pBABE-Puro (Panel A) or pBABE-Puro-hTERT (Panel B). G418-resistant clones were obtained from HFF3 infected with vector alone (V1, V2) or with pBABE-Puro-hTERT (T5, T9), and from HFF1 cells infected with vector alone (GV1, GV2) or with pBABE-Puro-hTERT (GT3, GT8). Telomerase activity in these G-418 resistant clones was assayed by TRAP as described in M & M. Lanes labeled with N, P, H are negative control with no cell extract, positive control with cell extract from a telomerase-positive HT-1080 cells, and heat-inactivated HT-1080 cell extract, respectively.

To address the relative role of oxidative stress and telomere attrition in cellular senescence, we asked whether ectopic expression of hTERT might be capable of restoring telomerase activity and extending the lifespan of G6PD-deficient fibroblasts. Normal (HFF3) and G6PD-deficient fibroblasts (HFF1) were infected with the retroviral construct, pBABE-Puro-hTERT, which expresses hTERT under the control of the LTR promoter, and G418-resistant clones were screened for the expression of telomerase activity. G418-resistant, telomerase-positive, clones from infected normal fibroblasts (T5 and T9), or G6PD-deficient fibroblasts (GT3 and GT8), were randomly selected for further study. As shown in Figure 1, G418-resistant cells from pBABE-Puro-hTERT-transfected normal and G6PD-deficient fibroblasts expressed similarly high telomerase activity that was comparable to that of a telomerase-positive fibrosarcoma cancer cell line (HT1080), whereas no telomerase activity was detected in G418-resistant cells transfected with the pBABE-Puro vector.

**Figure 2:**
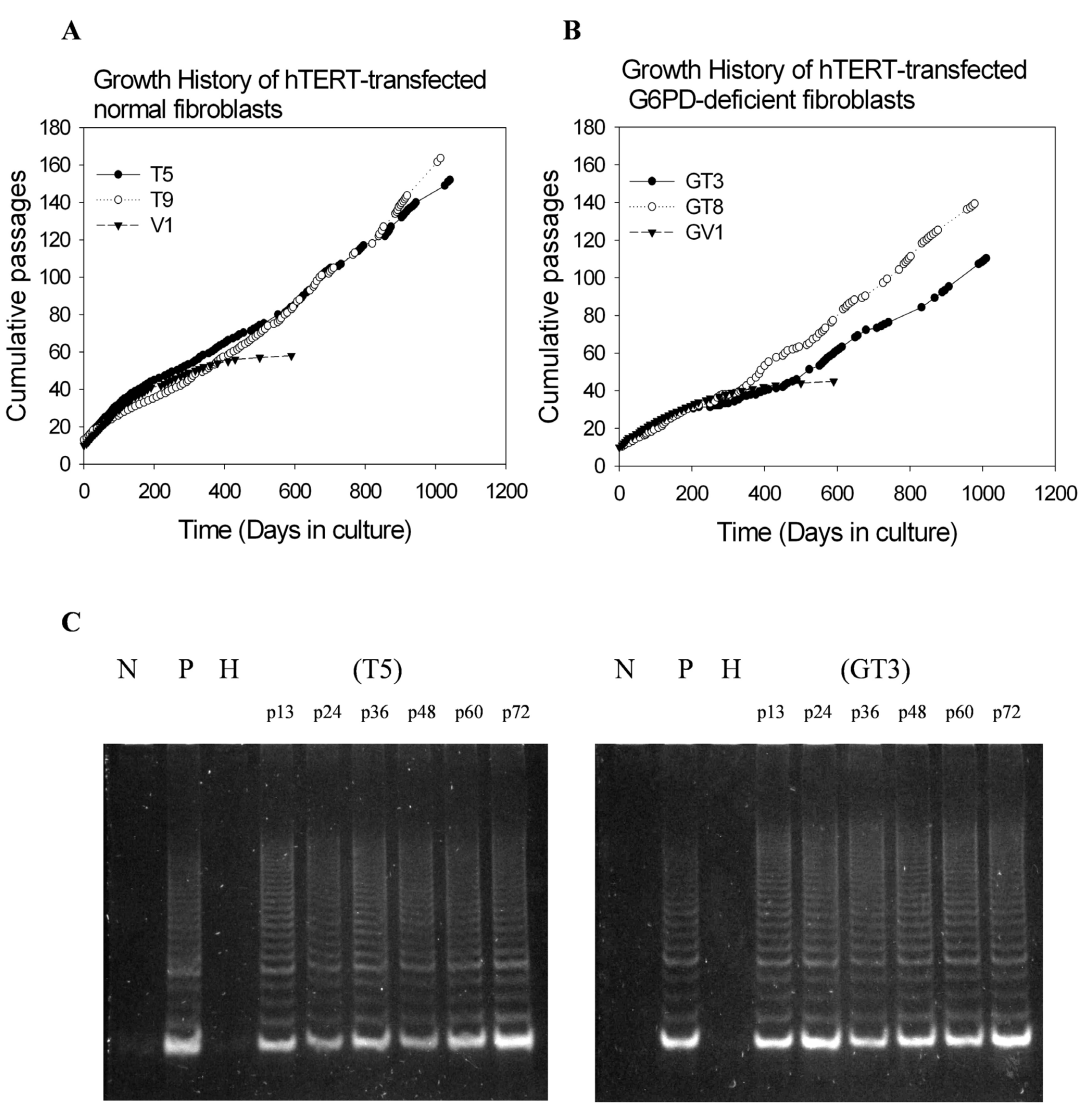
**Proliferation capability of hTERT-transfected normal and G6PD-deficient fibroblasts**. Normal fibroblasts HFF3 (Panel A) and G6PD-deficient fibroblasts HFF1 (Panel B) were infected with retroviral vector pBABE-Puro or pBABE-Puro-hTERT, and G418-resistant clones were obtained as described in legend to Fig. 1. These G418-resistant cells were cultivated by successive passages with 1:8 spliting. The telomerase activity of hTERT-transfected T5 and GT3 cells at various passages (p13 to p72) was determined by TRAP and shown in Panel C. Lanes labeled with N, P, H in Panel C are negative control with no cell extract, positive control with cell extract from a telomerase-positive HT-1080 cells, and heat-inactivated HT-1080 cell extract, respectively.

To determine whether ectopic expression of telomerase might be capable of extending the limited population doublings previously observed for normal and G6PD-deficient fibroblasts, we examined the proliferative capacity of hTERT-expressing fibroblasts. As shown in Fig. 2A and 2B, cells transfected with vector alone ceased to proliferate after about 55 and 40 passages for V1 and GV1, respectively. In contrast, hTERT-expressing fibroblasts derived from normal or G6PD-deficient patients continued to proliferate over 105 passages and showed no evidence of senescence, indicating that these cells were immortal. Accompanying the acquisition of unlimited growth potential in these fibroblasts was stable expression of telomerase activity, which persisted over the entire time of cell propagation (Fig. 2C).

### hTERT-immortalized G6PD-deficient fibroblasts do not exhibit a transformed phenotype

**Figure 3:**
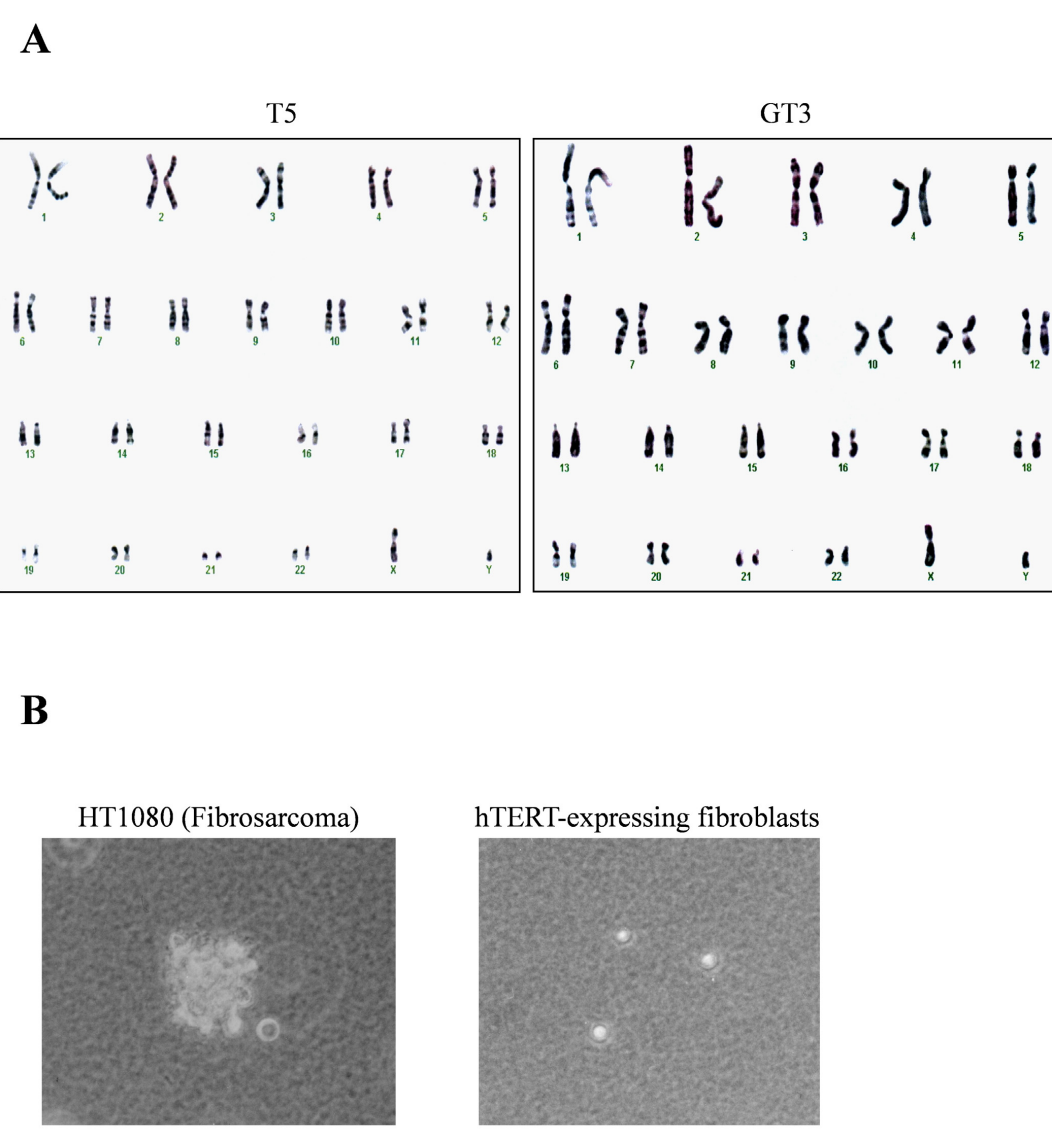
**The karyotypes and anchorage-independent growth potential of hTERT-expressing normal and G6PD-deficient fibroblasts**. The karyotypes of hTERT-expressing normal fibroblasts (T5) and G6PD-deficient fibroblasts (GT3) were determined as described in Materials and Methods; representative data are shown in panel A. Anchorage-independent growth was assayed by the ability to form colonies in soft agar. Representative results for hTERT-expressing G6PD-deficient fibroblasts are shown in panel B (right panel); no colonies formed and only single cells were detected. In HT1080 fibrosarcoma cell positive controls, colonies were detected (left panel).

To address the possibility that hTERT-immortalization of G6PD-deficient fibroblasts could induce changes associated with a transformed phenotype, we examined karyotype and anchorage-independent growth. Representative results of a karyotype analysis are shown for T5 and GT3 in Figure 3A. Both the hTERT-expressing normal and G6PD-deficient fibroblasts had a normal complement of diploid chromosomes (i.e., 46 + XY), and no abnormal chromosomal structures (e.g., translocations) were detected from a total of 20 cells analyzed in each preparation. To analyze anchorage-independent growth, we examined the ability of hTERT-expressing normal and G6PD-deficient fibroblasts to form colonies in soft agar (Fig. 3B). Whereas the fibrosarcoma HT1080 cells displayed 60% colony-forming efficiency, colony formation was not detected in hTERT-expressing normal or G6PD-deficient fibroblasts from six independent experiments with 5 × 10^3 ^cells plated in each experiment, indicating that these immortal cells do not acquire an anchorage-independent growth phenotype.

### Telomere length stabilizes after reaching a critical length in hTERT-expressing G6PD-deficient fibroblasts

**Figure 4:**
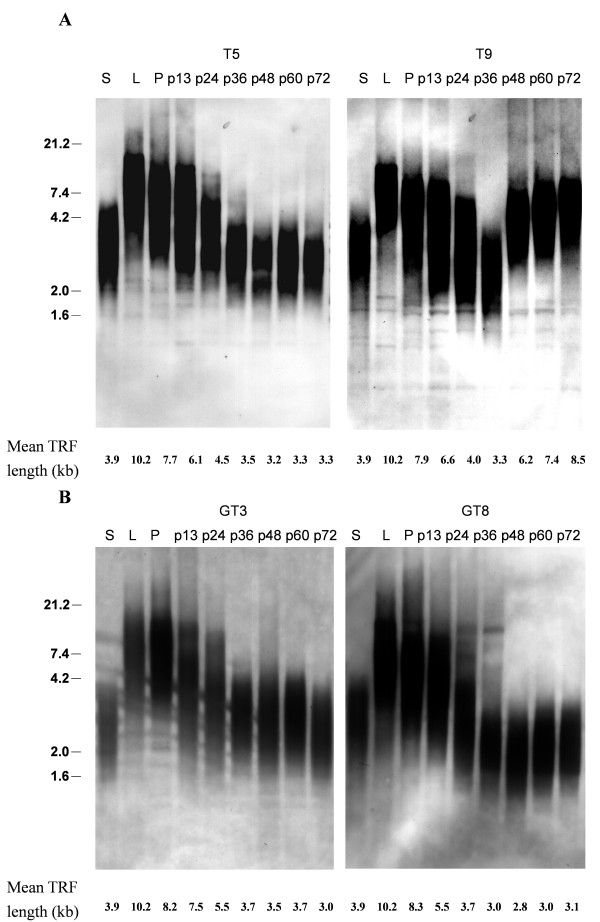
**Telomere lengths of hTERT-expressing normal and G6PD-deficient fibroblasts**. Cells were cultivated successively by 1:8 spliting in each passage. The telomere lengths of hTERT-expressing normal fibroblasts (Panel A) and G6PD-deficient fibroblasts (Panel B) at different passages were determined as described in M & M. Lanes S and L are controls supplied with the assay kit for DNAs having short or long TRF lengths, respectively. Lane P is the DNA from the parental cells before transfection. The mean telomere restriction fragment (TRF) lengths in kilobases (kb) are indicated below each lane. Molecular weight (M.W.) markers ranging from 1.6 to 21.2 kb are marked at the left.

Expression of telomerase is thought to counteract telomere attrition and thus provide escape from replicative senescence. To determine whether the ectopic expression of hTERT stabilizes telomere length in G6PD-deficient fibroblasts, we analyzed TRF lengths. As shown in Figure 4A, telomere length continued to decrease in hTERT-expressing normal fibroblasts, T5 and T9, in early-passage cells, despite the fact that these cells express high levels of telomerase activity. After passage 36, the TRF length in T5 cells appeared to stabilize at approximately 3.3 kb, whereas TRF length in T9 cells increased in later passages, increasing from ~3.3 kb to 6.2–8.5 kb. Both hTERT-expressing G6PD-deficient fibroblasts, GT3 and GT8, displayed a pattern of telomere length reduction and stabilization that was similar to that of normal T5 cells, with TRF decreasing in earlier-passage cells but then stabilizing at 3–3.7 kb after passage 36 (Fig. 4B).

### Resistance of hTERT-expressing G6PD-deficient fibroblasts to H2O2-induced premature senescence

**Figure 5:**
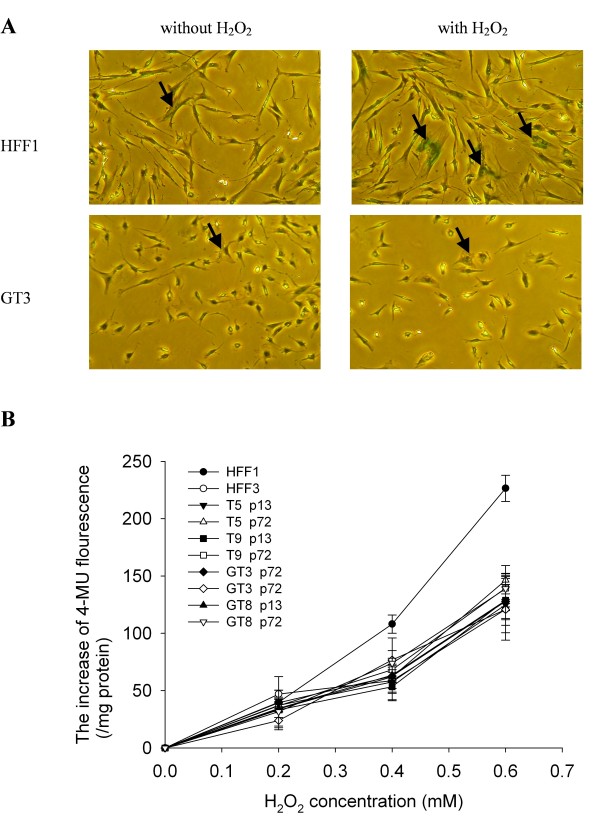
**H_2_O_2_-induced senescence in G6PD-deficient fibroblasts**. **A**. Cells were treated with 350 μM H_2_O_2 _for 1.5 h and then cultured for another 72 h before staining for senescence-associated β-galactosidase (SA-β-Gal) activity. Representative results for HFF1 and GT3 cells with or without H_2_O_2 _treatment are shown in the right and left panels, respectively. Examples of cells that are positive for SA-β-Gal staining are indicated by arrow. In the quantification, any cell that has detectable green staining was scored as positive for SA-β-Gal. **B**. Cells were treated with different concentrations of H_2_O_2 _for 1.5 h and then cultured for another 72 h. Quantification of premature senescence was determined by the rate of conversion of 4-methylumbellliferyl-β-D-galactopyranoside (MUG) to fluorescent product 4-methylumbelliferone (4-MU) as described in M & M. Both the early passage (p13) and late passage (p72) cells of T5, T9, GT3 and GT8 were included in this study. Data are the means ± SD from 4 independent experiments.

To address the mechanism by which ectopic expression of hTERT enables G6PD-deficient cells to overcome premature senescence, we measured the ability of hTERT-expressing cells to cope with oxidative stress induced by treatment with exogenous H_2_O_2_. As shown in Figure 5A, under normal culture conditions there were very few cells among G6PD-deficient HFF1 fibroblasts (passage 22) and hTERT-expressing G6PD-deficient GT3 fibroblasts (passage 72) that were positive for SA-β-Gal staining (less than 5%). Treatment with H_2_O_2 _increased the number of SA-β-Gal-positive cells in both the HFF1 and GT3 populations, indicating that H_2_O_2_induces premature senescence in both cells. Interestingly, we noted that there is a greater increase of SA-β-Gal-positive cells in the H_2_O_2_-treated HFF1 cells (55 ± 9%) than in the H_2_O_2_-treated GT3 cells (21 ± 1.4%). To confirm the significance of this observation, we treated parental and hTERT-expressing fibroblasts from both normal and G6PG-deficient patients with different concentrations of H_2_O_2 _and quantified the extent of H_2_O_2_-induced premature senescence. As shown in Figure 5B, the levels of H_2_O_2_-induced premature senescence in the early passage (p13) and late passage (p72) cells of T5, T9, GT3 and GT8 were similar to HFF3, but were significantly greater in HFF1 cells. These results indicate that ectopic expression of hTERT in G6PD-deficient cells renders the cells more resistant to H_2_O_2_-induced premature senescence.

## Discussion

The accelerated cellular senescence characteristic of G6PD-deficient fibroblasts would seem to represent a clear case of a senescence mechanism based on accumulated oxidative DNA damage rather than one involving accelerated telomere attrition [26]. This mechanistic transparency makes these cells an ideal system for addressing the relative role of oxidative stress and telomere attrition in cellular senescence. Somewhat surprisingly, we found that ectopic expression of hTERT prevented the accelerated senescence of G6PD-deficient cells and led to their immortalization (Fig. 2). The growth rate of hTERT-expressing G6PD-deficient fibroblasts, however, remained slower than that of hTERT-expressing normal fibroblasts (Fig. 2A and 2B), as noted previously (25). This finding suggests that hTERT overexpression may attenuate senescence induction by oxidative stress, but does not suppress the growth defect caused by G6PD-deficiency. To test this hypothesis, we measured the ability of hTERT-expressing G6PD-deficient cells to cope with H_2_O_2_-induced oxidative stress. Similar to results reported by others [31], we found no difference in stress-induced premature senescence (SIPS) between normal and hTERT-expressing normal fibroblasts (Fig. 5). However, hTERT-expressing G6PD-deficient cells became more resistant to H_2_O_2_-induced premature senescence (Fig. 5), indicating that the increased sensitivity to oxidative stress in G6PD-deficient cells is prevented by the expression of hTERT.

Ectopic expression of hTERT has also been shown previously to immortalize fibroblasts derived from individuals with Ataxia telangiectasia (A-T), Nijimegen breakage syndrome (NBS), Hutchinson-Gilford progeria syndrome (HGPS), and Werner Syndrome (WS) [32-36]. Although the genetic defects in A-T, NBS, HGPS, WS, and G6PD-deficiency patients are very different, fibroblasts derived from these individuals have one common phenotype: they all undergo accelerated senescence *in vitro *[25,34,36,37]. The premature senescence of mitotic cells derived from A-T, HGPS, and NBS patients has been correlated with an increased rate of telomere loss [31,34,38], whereas the mechanism responsible for the premature senescence of WS and G6PD-deficient fibroblasts appears to be different and has been postulated to reflect the accumulation of DNA damage [26,38]. The fact that ectopic expression of hTERT immortalizes fibroblasts derived from individuals with any of these different defects indicates that telomerase may attenuate senescence induction triggered by either telomere attrition or genome-wide DNA damage. Ectopic expression of hTERT has also been shown to increase radioresistance of adult human mesenchymal stem cells [39], to circumvent hyperglycemia-induced premature senescence [40], and to prevent apoptosis induced by tumor necrosis factor [41]. These observations suggest that expression of hTERT not only activates telomere maintenance but may also affect signaling that protects cells from oxidative stress and other stimuli. Indeed, increasing evidence is emerging to implicate that hTERT has functions beyond telomere maintenance [42].

hTERT-immortalization of cells is generally attributed to the activation of telomerase activity and the subsequent counteraction of telomere attrition. Surprisingly, we observed that telomere lengths continued to decrease in earlier-passage cells from both hTERT-expressing normal and G6PD-deficient fibroblasts, despite the fact that these cells expressed high levels of telomerase activity (Fig. 1). In fact, we observed that TRF lengths from one hTERT-expressing normal cell line (T5) and two hTERT-expressing G6PD-deficient cell lines (GT3 and GT8) were shortened to ~3–3.7 kb before stabilization (Fig. 4). The parental fibroblasts used for the derivation of these hTERT-expressing cells undergo senescence when TRF lengths decrease to approximately 5–6 kb [25], suggesting that dysfunctional telomeres may already have formed when the TRF length was reduced to 5–6 kb in normal fibroblasts. However, senescence was not induced in these hTERT-expressing cells even when the TRF lengths were reduced to less than 5 kb, suggesting that hTERT, in addition to serving as a subunit for telomerase, acts by some other mechanism to prevent senescence induction. Shortening of telomere lengths in the presence of telomerase activation has also been observed by others [28,43], and a protective role for hTERT in telomere capping has been suggested [28,44]. In addition, hTERT has been found to associate with human telomeres, and ectopic expression of hTERT causes transcriptional alterations in a subset of genes; this may lead to increased genomic stability and enhanced DNA repair activity [45]. At this time, the molecular details of hTERT involvement in senescence induction remain obscure.

It is currently not known why activation of telomerase does not result in an immediate stabilization of telomere lengths. Presumably, telomere length is influenced not only by telomerase levels but also depends on a control pathway that acts in *cis *at each individual telomere [46]. Several telomere-binding proteins, such as TRF1, TRF2, and Pot1, are known to participate in telomere-length regulation and chromosome-end protection [46]. It is likely that some of these factors prevent telomerase from functioning at the telomere until telomeres are shortened to a critical length. Our finding that the lengths of telomeres in hTERT-expressing cells were reduced to approximately 3–3.7 kb before stabilization (Fig. 4) provides support for this notion.

In conclusion, we have shown that ectopic expression of hTERT immortalizes fibroblasts derived from normal and G6PD-deficient patients. The accelerated cellular senescence observed in G6PD-deficient cells has been shown to be attributable to increased accumulation of oxidative DNA damage [26]. As this oxidant-induced senescence was overridden by the ectopic expression of hTERT, we suggest that hTERT, in addition to providing a subunit for telomerase, may also function in regulating senescence induction.

## Competing interests

The authors declare that they have no competing interests.

## Authors' contributions

MC and HH participated in the preparation of retroviral particles and isolation of hTERT-expressing clones, YW carried out the rest of research, DTC and TVW conceived of the study and design research, YW and TVW wrote the paper. All authors read and approved the final manuscript.
